# The interrelationships between Src, Cav-1 and RhoGD12 in transitional cell carcinoma of the bladder

**DOI:** 10.1038/bjc.2012.52

**Published:** 2012-02-21

**Authors:** T Qayyum, G Fyffe, M Duncan, P A McArdle, M Hilmy, C Orange, G Halbert, M Seywright, P G Horgan, M A Underwood, J Edwards

**Affiliations:** 1 Institute of Cancer, College of MVLS, University of Glasgow, Western Infirmary, Glasgow G11 6NT, UK; 2 Department of Urology, Royal Infirmary, Glasgow G4 0SF, UK; 3 Department of Pathology, Western Infirmary, Glasgow G11 6NT, UK; 4 School of Medicine, College of MVLS, University of Glasgow, Royal Infirmary, Glasgow G4 0SF, UK

**Keywords:** Src kinase, Cav-1, RhoGD12, bladder cancer, survival

## Abstract

**Background:**

The aim of this current study was to assess the expression and activity of Src family kinases, focal adhesion kinase (FAK), caveolin (Cav-1) and RhoGD12 in bladder cancer.

**Methods:**

Fifty-eight patients with a new diagnosis of bladder cancer undergoing transurethral resection were included. Immunohistochemical staining was utilised to assess expression of c-Src, dephosphorylated (SrcY^530^), phosphorylated Src (Y^419^), phosphorylated FAK (FAK Y^861^), Cav-1 and RhoGD12. Expression was assessed using the weighted histoscore method.

**Results:**

High expression of dephosphorylated Y^527^, phosphorylated Y^416^ and phosphorylated FAK Y^861^ in the membrane were associated with increased cancer-specific survival (*P*=0. 01, *P*=0.001, *P*=0.008, respectively) and expression of Y^416^ in the membrane was an independent factor on multivariate analysis when combined with known clinical parameters (*P*=0.008, HR 0.288, 95% CI 0.11–0.72).

**Conclusion:**

These results demonstrate that in contrast to other solid tumours, activation of the Src family members and downstream signalling proteins are associated with a good prognosis in transitional cell carcinoma of the bladder, and activated Src has a positive relationship with RhoGD12.

Worldwide, there are around 360 000 new cases of bladder cancer diagnosed each year (www.cancerresearchuk.org). In the UK alone, bladder cancer is the fifth most common solid non-cutaneous malignancy with approximately 10 000 new cases and 5000 deaths annually (www.cancerresearchuk.org). Overall survival is variable; those with non-muscle invasive disease have an 80–90% 5-year survival, whereas in those with muscle invasive disease, the 3-year survival is between 25–50% depending on the degree of invasive disease (www.cancerresearchuk.org).

In those with non-muscle invasive disease, the recurrence and progression rate is strongly associated with tumour stage and grade. These factors can be categorised to show the likelihood for recurrence and progression using EORTC risk tables (Sylvester *et al*, 2006). Even in those considered low risk, the 5-year recurrence is 31%, increasing to 78% in those considered to be in the risk high category (Sylvester *et al*, 2006). The 5-year progression rate in those at high risk is 45% (Sylvester *et al*, 2006).

In those with muscle invasive disease undergoing radical cystectomy, the 5-year survival is only 50% (Dalbagni *et al*, 2001; Stein *et al*, 2001; Stein and Skinner, 2006). Despite neoadjuvant chemotherapy in this patient group, published data has only shown a 5% absolute improvement in survival at 5 years (Advanced Bladder Cancer (ABC) Meta-analysis Collaboration, 2005). The role of neoadjuvant radiotherapy has shown a significant increase in survival in randomised studies (Slack *et al*, 1977; Smith Jr *et al*, 1997). For those with non-resectable disease, palliation of symptoms is the only treatment option.

One potential molecular target is the non-receptor tyrosine kinase Src, the first identified human proto-oncogene. Src kinase has a role in signal transduction of multiple oncogenic cellular processes, including migration, adhesion, invasion, angiogenesis, proliferation and differentiation, and has significant interactions with other cellular proteins such as growth factor receptors (Kopetz *et al*, 2007). The c-Src (commonly referred to as Src) is the prototypical member of the Src kinase family (SFK), with a total of eight members expressed in mammalian cells (c-Src, Blk, Fgr, Fyn Yes, Hck, Lck and Lyn). Src is composed of a C-terminal tail, kinase domain, two protein–protein interaction domains (SH2, SH3) and a unique amino-terminal domain that varies between the Src family members. Src is activated by a number of pathways. Src kinase activation involves dephosphorylation of a conserved tyrosine residue in the C-terminal negative regulatory tail region (Y^530^) and subsequent autophosphorylation of the Y^419^ site in the kinase domain (Cooper and Howell, 1993; Engen *et al*, 2008). Consequently, antibodies to the dephosphorylation of Y^530^ and phosphorylation of Src kinase at the Y^419^ sites can be used as markers for activated Src kinase (Campbell *et al*, 2008). Activated Src kinase translocates to the cell membrane (Frame, 2002), and therefore, cellular location may also be employed as a surrogate marker of activation (Luo *et al*, 2008). Also, when Src is activated, several downstream signalling proteins such as focal adhesion kinase (FAK) are phosphorylated and could therefore act as biomarkers for Src activation (Nam *et al*, 2005). FAK is phosphorylated at several sites by Src such as Y^397^, Y^576^ and Y^577^, but it has been reported that Y^861^ is the major site in the carboxyl-terminal domain of FAK (Schaller *et al*, 1994; Calalb *et al*, 1995, 1996). It has been shown that Src expression and activity decreases with bladder tumour stage (Fanning *et al*, 1992; Blaveri *et al*, 2005; Sanchez-Carbayo *et al*, 2006; Wu *et al*, 2009; Thomas *et al*, 2011), although a positive correlation has been shown with breast and prostate cancers (Elsberger *et al*, 2009; Tatarov *et al*, 2009).

Caveolin-1 (Cav-1) is the major coat protein of caveolae, regulating the activity of signalling molecules in cancer (Liu *et al*, 2002), and can make both a negative and positive impact on cancer progression. Cav-1 has been implicated in preventing cell transformation (Galbiati *et al*, 1998) and promoting cell cycle arrest and senescence (Galbiati *et al*, 2001; Volonte *et al*, 2002). Cav-1 expression has been shown to be elevated in various malignancies such as colon (Fine *et al*, 2001), thyroid (Ito *et al*, 2002), lung (Ho *et al*, 2002) and bladder (Fong *et al*, 2003) cancers. In bladder cancer, it has previously been reported that Cav-1 expression is elevated at the onset of oncogenesis (Fong *et al*, 2003) and these levels rise further with progression of the disease in terms of stage and grade (Rajjayabun *et al*, 2001; Fong *et al*, 2003; Kunze *et al*, 2006). Caveolin-1 expression has been demonstrated to be associated with increased cytoplasmic expression in colon cancer but was not associated with tumour stage (Fine *et al*, 2001), whereas membranous expression in ovarian cancer was associated with shorter survival (Davidson *et al*, 2001). Cav-1 was first described as the major substrate for Src in v-Src-transformed cell lines (Glenney Jr and Zokas, 1989; Lee *et al*, 2000), and phosphorylated Cav-1 can either inhibit Src through recruitment of the C-terminal (Li *et al*, 1996; Cao *et al*, 2002) or promote Src activation through an unknown mechanism (Grande-Garcia *et al*, 2007). In addition, high expression of Cav-1 and low expression of Src were associated with metastasis and poor survival in bladder cancer and suggested that these both control bladder metastases through the Rho–ROCK pathway (Thomas *et al*, 2011).

RhoGD12 is known to have a negative and positive impact on cancer. It has previously been demonstrated that RhoGD12 positively correlates with tumour progression and metastases in gastric, ovarian and breast cancers (Tapper *et al*, 2001; Zhang and Zhang, 2006; Cho *et al*, 2009), whereas others have reported it to be a metastatic suppressor in malignancies, including prostate, breast, lung and bladder cancers (Gildea *et al*, 2002; Theodorescu *et al*, 2004; Moon *et al*, 2010; Niu *et al*, 2010). There is controversy in the literature regarding RhoGD12 expression; it has been reported to be both more prominent in the nuclei and cytoplasm of tumour cells (Theodorescu *et al*, 2004; Cho *et al*, 2009). Interestingly, a negative relationship was observed between RNA expression of Src and RhoGD12; it was further suggested that membrane expression of RhoGD12 is elevated when co-transfected with Src Y^527^ (Wu *et al*, 2009). Given that Src is still inactive in this state, this would suggest that RhoGD12 expression has a positive relationship with Src inactivity and not Src activity as previously reported.

We hypothesise that it is the activity of Src that drives the relationship with Cav-1 and RhoGD12 expression. The aims of this study were to assess expression of Src, Cav-1 and RhoGD12 in relationship to cancer-specific survival, and to examine the potential interrelationships between these markers.

## Patients and methods

Patients with organ-confined bladder cancer were included for this study. All patients had transitional cell carcinoma. These patients had undergone resection based on investigations in the North Glasgow NHS Trust. Only those patients were included, whose initial CT scans at the time of diagnosis showed no evidence of regional or metastatic disease, patients who had undergone instillation of intravesical chemotherapy post operation, and whose only subsequent treatment was transurethral resection of recurrences and intravesical therapy. Those patients that subsequently underwent further treatment by way of radical surgery or radiotherapy were excluded. Patients were staged pathologically according to the TNM classification and graded according to the World Health Organisation/International Society of Urological Pathology criteria (Epstein *et al*, 1998). Cancer-specific survival rate was the time from diagnosis until time of death or last follow-up. The cause of death was determined by linkage through the Scottish Cancer Registry. In those who were deceased, if the primary cause of death was of bladder cancer, these were classed as cancer specific, and all other causes were non-cancer specific deaths. The Research Ethics Committee of West of Scotland has approved the study.

Immunohistochemical staining was utilised to assess the expression of c-Src, dephosphorylated Src at Y^530^, phosphorylated Src at Y^419^, FAK at Y^861^, Cav-1 and RhoGD12. Both antibodies for dephosphorylated Src (Y^530^) and phosphorylated Src (Y^419^) are not specific for c-Src because of sequence homogeneity between family members, and can therefore also detect other family members, including Fyn, Yes and Fgr.

Src kinase and activated Src kinase expression (c-Src and Src Y^419^) were investigated using antibodies for c-Src (36D10, Cell Signalling Technology, Beverly, MA, USA) and Y^416^Src (Cell Signalling Technology). Dephosphorylated Src and phosphorylated FAK were investigated using antibodies for Src dephosphorylated at Y^527^ (dephosphorylated Y^527^) and FAK Y^861^ (Invitrogen, Paisley, UK). In humans, the activated phosphorylations that were investigated in the current study are amino acids Y^530^ and Y^419^. Antibodies used relate to the rabbit sequence and not the human sequence. Expression for Cav-1 and RhoGD12 were investigated using an anti-Caveolin 1 antibody (Abcam, Cambridge, UK) and D4-GDI (Spring Bioscience, Pleasanton, CA, USA), respectively.

Tissue sections were dewaxed and rehydrated through graded alcohol. Antigen retrieval was performed by heating tissue sections under pressure for 5 min in a pressure cooker, using citrate buffer pH 6 for c-Src, dephosphorylated Y^527^, FAK Y^861^, Cav-1, RhoGD12 and EDTA buffer pH 9 for Src Y^416^. Endogenous peroxidase activity was blocked by incubation in 3% hydrogen peroxide (H_2_O_2_). To reduce non-specific binding, the tissue sections were then incubated with 5% normal horse serum (Vector Laboratories, Burlingame, CA, USA) in antibody dilutent (DAKO Cytomation, Glostrup, Denmark) for 20 min at room temperature. Incubation with primary antibody was performed with c-Src (1 : 200) for 60 min at room temperature and overnight at 4 °C for antibodies for dephosphorylated Src Y^527^ (1 : 3000), phosphorylated Src Y^416^ (1 : 25), FAK Y^861^ (1 : 200), Cav-1 (1 : 500) and RhoGD12 (1 : 200).

Signal was amplified and visualised using the DAKO Envision Kit (DAKO Cytomation) and the chromagen 3,3′-diaminobenzidine (Vector Laboratories).

Sections were counterstained, dehydrated and mounted. In each run, a positive and negative isotype-matched control was included to ensure no false-positive staining.

### Scoring

Protein expression was assessed using the weighted histoscore method (H score method; Kirkegaard *et al*, 2006). The weighted histoscore grades staining intensity as negative (0), weak (1), moderate (2) and strong (3), then multiplication of the percentage of tumour cells within each category. Agreement between observers was excellent and measured in interclass correlation coefficient, respectively.

Statistical analysis was undertaken using SPSS (Chicago, IL, USA). Cancer-specific survival rates were generated using the Kaplan–Meier method. The log rank test was utilised to compare significant differences between subset groups using univariate analysis. Multivariate analysis was carried out based on the results of the univariate analysis. Multivariate Cox regression analysis was performed to identify those factors that were independently associated with cancer-specific death. A stepwise backward procedure was utilised to ascertain which of the variables had a significant independent relationship with survival. A *χ*^2^-analysis was utilised to assess relationships between pathological parameters and the biomarkers at the various locations. Pearson's correlation was utilised to assess if relationships could be identified between the various proteins at the various cellular locations. *P*-values <0.0003 were deemed significant according to Bonferronis correction.

## Results

Analysis was based on 58 bladder transitional cell carcinoma patients with full clinical follow-up. Median age at diagnosis was 69 years (range 43–91). Median follow-up was 33 months (range 1–180). In all, 27 patients died of their disease.

**Figure 1:**
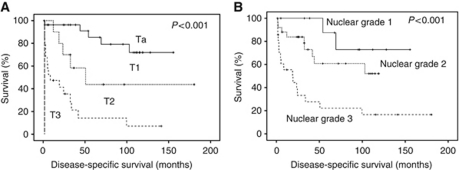
(**A**) Shows a Kaplan–Meier plotted for T-stage against cancer-specific survival, log rank test *P*<0.001. (**B**) Shows a Kaplan–Meier plotted grade against cancer-specific survival, log rank test *P*<0.001.

Initial analysis was based on known clinico-pathological features, which are known to be prognostic indicators for survival in bladder cancer. T-stage (Figure 1A) and tumour grade (Figure 1B) were significantly associated with poor prognosis, thus validating our cohort for use in a biomarker study (Table 1).

**Figure 2:**
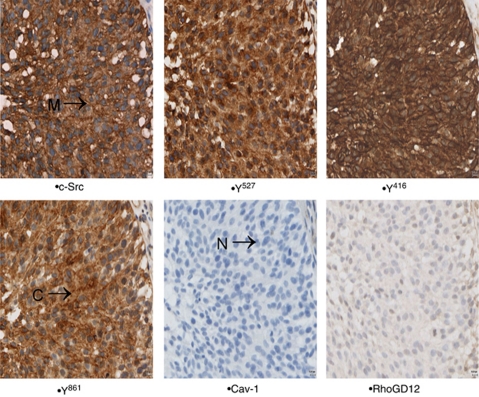
Shows representative images of immunohistochemistry for bladder cancer of Src kinase, Src Y^527^, Src Y^416^ and FAK Y^861^. Membrane staining denoted by M, cytoplasmic staining by C and nuclear staining N.

Each cellular location was independently assessed for expression levels (Figure 2). Tumours were divided into those with high (above median) or low (below or equal to the median) expression.

### c-Src kinase

Of the tumours investigated, 74% showed some nuclear expression, 100% cytoplasmic expression, and 93% membrane expression. Tumours were subdivided into with high (above median) and low (below median) expression. The *χ*^2^-analysis demonstrated that membrane c-Src negatively correlated with tumour grade (*P*=0.024, Table 2), but no correlation was demonstrated with age, T-stage, recurrence or metastases. These results suggested that when located in the membrane, c-Src conferred a good prognosis; however, on univariate analysis, expression of c-Src at the different cellular locations did not show significance (Table 1), therefore no correlation with cancer-specific survival was observed for this marker. Pearson's correlation demonstrated that membrane c-Src expression positively correlated with cytoplasmic c-Src (*P*<0.0001, Table 3).

### Y^527^ Src kinase

Of the tumours investigated, 3% showed some degree of nuclear expression, 98% cytoplasmic expression, and 93% membrane expression. The *χ*^2^-analysis demonstrated that expression of membrane-dephosphorylated Y^527^ negatively correlated with T-stage and grade (*P*=0.021, *P*=0.011, Table 2), but no correlation was demonstrated with age, recurrence or metastases. On univariate analysis, high expression of membrane-dephosphorylated Y^527^ was associated with increased cancer-specific survival (*P*=0.01, Table 1). In contrast, high expression of nuclear-dephosphorylated Y^527^ was associated with decreased cancer-specific survival, *P*=0.008; however, this result should be viewed with caution, as only 3% of tumours exhibited high nuclear-dephosphorylated Y527 expression, (Table 1). Pearson's correlation demonstrated expression of membrane Y^527^ positively correlated with membrane c-Src expression (*P*<0.0001, Table 3), suggesting that both surrogate markers of c-Src activation correlate with each other, and therefore validating their use in this manner.

### Y^416^ Src kinase

**Figure 3:**
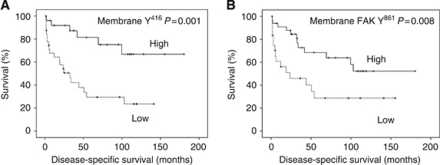
(**A**) Shows a Kaplan–Meier plotted for high and low membrane Y^416^ expression against cancer-specific survival, log rank test *P*=0.001. (**B**) Shows a Kaplan–Meier plotted for high and low membrane FAK Y^861^ expression against cancer-specific survival, log rank test *P*=0.008.

Of the tumours investigated, 90% showed some degree of nuclear expression, 100% cytoplasmic expression, and 100% membrane expression. The *χ*^2^-analysis demonstrated that expression of membrane Y^416^ negatively correlated with tumour grade and evidence of metastasis at follow-up (*P*=0.005, *P*=0.001, Table 2), but no correlation was demonstrated with age, T-stage and recurrence. On univariate analysis, high expression of membrane Y^416^ was associated with increased cancer-specific survival (*P*=0.001, Table 1, Figure 3A) and was shown to be an independent factor on multivariate analysis (*P*=0.008, HR 0.288, CI 0.11–0.72, Table 1).

### FAK Y^861^

Of the tumours investigated, 10% showed some degree of nuclear expression, 34% cytoplasmic expression, and 91% membrane expression. The *χ*^2^-analysis demonstrated that expression of membrane Y^861^ negatively correlated with recurrence and evidence of metastases (*P*=0.026, *P*=0.045, Table 2), but no correlation was demonstrated with age, T-stage or grade. On univariate analysis, high expression of membrane Y^861^ was associated with increased cancer-specific survival (*P*=0.008, Table 1, Figure 3B). Pearson's correlation demonstrated the expression of cytoplasmic FAK Y^861^ demonstrated a positive correlation with cytoplasmic Y^527^ (*P*<0.0001, Table 3). Expression of membrane FAK Y^861^ demonstrated a positive correlation with membrane c-Src, Y^527^ and Y^416^ (*P*<0.0001, *P*<0.0001, *P*=0.0001, Table 3). Therefore, all markers of Src activation correlated with activation of the downstream marker FAK Y^861^.

### Caveolin-1

Of the tumours investigated, 2% showed degree of nuclear expression, 97% cytoplasmic expression, and 24% membrane expression. The *χ*^2^-analysis demonstrated that expression of membrane Cav-1 positively correlated with tumour grade (*P*=0.026, Table 2), but no correlation was demonstrated with age, T-stage, recurrence or metastases. On univariate analysis, however, high expression of nuclear Cav-1 was associated with decreased cancer-specific survival (*P*=0.042, Table 1), but as only 2% of tumours exhibited high nuclear expression, these results need to be viewed with caution.

### RhoGD12

Of the tumours investigated, 91% showed degree of nuclear expression, 91% cytoplasmic expression, and 71% membrane expression. The *χ*^2^-analysis demonstrated that expression of nuclear RhoGD12 had a negative correlation with metastases (*P*=0.036, Table 2), but no correlation was demonstrated with age, T-stage, grade or recurrence. Pearson's correlation demonstrated expression of cytoplasmic RhoGD12 demonstrated a positive correlation with nuclear RhoGD12 (*P*<0.0001, Table 3). Expression of membrane RhoGD12 demonstrated a positive correlation with cytoplasmic and membrane c-Src (*P*<0.0001, *P*<0.0001, Table 3) and cytoplasmic RhoGD12 expression (*P*<0.0001, Table 3). This suggests that RhoGD12 expression is associated with levels of c-Src in bladder cancer.

## Discussion

To our knowledge, this is the only study investigating the role of Src kinase, dephosphorylation status (Y^530^), autophosphorylation status (Y^419^), the downstream signalling protein FAK Y^861^, Cav-1 and RhoGD12 expression in one cohort of bladder cancers.

This study demonstrates that when c-Src is active (utilising membrane expression as a surrogate marker of activation), an inverse correlation with tumour grade is observed. These results are consistent with those that have previously reported a negative correlation of Src expression with bladder aggressiveness (Fanning *et al*, 1992; Blaveri *et al*, 2005; Sanchez-Carbayo *et al*, 2006; Wu *et al*, 2009; Thomas *et al*, 2011), and further reiterates that Src inhibitors should be utilised with much caution for cancer prevention in transitional cell carcinoma.

It has been suggested that a biomarker for prediction of Src kinase activity would be to measure phosphorylation of the protein at a site associated with activity (Bolen *et al*, 1987; Masaki *et al*, 1998), and Src kinase, when active, translocates to the membrane (Frame, 2002). When examining the expression of various phosphorylated statuses of Src, we demonstrated that expression of dephosphorylated Src (dephosphorylated Y^527^) and autophosphorylated Src (Y^416^) negatively correlated with tumour grade. Furthermore, increased expression of membrane Src Y^416^ was an independent predictor of improved cancer-specific survival. The antibody for Y^419^ is not specific for one particular Src family member, but because of sequence homogeneity, cross reacts with all Src family members that are phosphorylated at this site. Therefore, as c-Src itself in this study is not associated with clinical outcome measures, we hypothesise that expression of another of the SFK members is driving the association with improved outcome observed in the current study. Further evidence supporting the observation that activation of Src family members confers an improved prognosis is by assessing the relationships of the downstream signalling protein FAK Y^861^ to outcome. Expression of FAK Y^861^ was observed to be negatively associated with recurrence, presence of metastases and increased cancer-specific survival.

Cav-1 expression can have both a positive and negative effect on cancer progression. In this study, when assessing expression of Cav-1, it was shown that presence in the membrane positively correlated with nuclear grade. This is also in keeping with previous work which has shown that Cav-1 expression was associated with grade (Rajjayabun *et al*, 2001; Fong *et al*, 2003; Kunze *et al*, 2006). It has also been demonstrated that Cav-1 expression was more membranous in those with ovarian cancer and had short-term survival (Fine *et al*, 2001), which is a similar finding to what we have shown that membranous expression has a positive correlation with grade, and this factor is associated with poorer survival. Previous work has shown that Cav-1 and Src have a reciprocal relationship in bladder cancer (Thomas *et al*, 2011). In this study, no such relationship reached significance, but it was demonstrated that Cav-1 and c-Src had a positive correlation and Cav-1 had a negative correlation with Y^416^. This also suggests that it is another of the SFK member, which is associated with a reciprocal relationship with Cav-1. It has been suggested that Cav-1 inhibits Src through recruitment of the C-terminal (Li *et al*, 1996; Cao *et al*, 2002), but in this study, there was no correlation between Cav-1 and Y^527^.

RhoGD12 expression has been thought of as being a suppressor of cancer progression and metastases, but has also been shown to positively correlate with cancer progression in various malignancies (Tapper *et al*, 2001; Zhang and Zhang, 2006; Cho *et al*, 2009). We have observed that nuclear expression of RhoGD12 negatively correlated with the presence of metastases at follow-up, which is consistent with work showing that RhoGD12 is a metastases suppressor in bladder cancer (Gildea *et al*, 2002; Theodorescu *et al*, 2004). Previous work has reported that expression of Src and RhoGD12 confers improved prognosis in bladder cancer (Fanning *et al*, 1992; Theodorescu *et al*, 2004; Blaveri *et al*, 2005; Sanchez-Carbayo *et al*, 2006; Thomas *et al*, 2011). It has also been reported active Src (Y^530^) results in elevated levels of RhoGD12 (Wu *et al*, 2009). Given that, when Src is dephosphorylated at Y^530^, it is not active yet, and this would suggest a relationship between inactive Src and RhoGD12. This study does demonstrate that expression of RhoGD12 is associated with less likelihood of metastases, but also shows a positive relationship between RhoGD12 and membrane c-Src. As c-Src is active when located in the membrane, we have therefore shown that it is active c-Src that has a relationship with RhoGD12.

This current study further reinforces that expression of Cav-1 confers poor prognosis. This study reports that RhoGD12 confers improved prognosis, but has a positive correlation with active c-Src. It has also been demonstrated that expression of dephosphorylated Src (Y^527^), autophosphorylated Src (Y^416^) and the downstream marker FAK Y^861^ confer improved cancer-specific survival. Furthermore, expression of Src Y^416^ is an independent factor associated with increased cancer-specific survival, suggesting that expression of another SFK member other than Src itself confers improved prognosis.

## Figures and Tables

**Table 1:** Impact of clinicopathological factors and protein expression/activation on patient survival

		**Univariate analysis**	**Multivariate analysis**	
	**Numbers**	***P*-value**	***P*-value**	**HR**
Age (<60/>60)	23/35	0.287		
T-stage (Ta/T1/T2/T3/T4)	27/20/10/1/0	**<0.001**	**<0.001**	3.22 (1.97–5.24)
Grade (1/2/3)	11/25/22	**<0.001**	—	—
c-Src nuc (negative/positive)	37/21	0.418	—	—
c-Src cyto (negative/positive)	31/27	0.308	—	—
c-Src mem (negative/positive)	30/28	0.22	—	—
Y^527^ Src nuc (negative/positive)	56/2	**0.008**	—	—
Y^527^ Src cyto (negative/positive)	37/21	0.405	—	—
Y^527^ Src mem (negative/positive)	26/32	**0.01**	—	—
Y^416^ Src nuc (negative/positive)	30/28	0.647	—	—
Y^416^ Src cyto (negative/positive)	29/29	0.264	—	—
Y^416^ Src mem (negative/positive)	33/25	**0.001**	**0.008**	0.288 (0.11–0.72)
Y^861^ FAK nuc (negative/positive)	52/6	0.995	—	—
Y^861^ FAK cyto (negative/positive)	38/20	0.732	—	—
Y^861^ FAK mem (negative/positive)	25/33	**0.008**	—	—
Cav nuc (negative/positive)	57/1	**0.042**	—	—
Cav cyto (negative/positive)	31/27	0.689	—	—
Cav mem (negative/positive)	44/14	0.177	—	—
Rho nuc (negative/positive)	29/29	0.186	—	—
Rho cyto (negative/positive)	30/28	0.712	—	—
Rho mem (negative/positive)	31/27	0.425	—	—

Abbreviations: Cav=caveolin; cyto=cytoplasmic; FAK=focal adhesion kinase; HR=hazards ratio; mem=membrane; nuc=nuclear.

Each clinical and pathological parameter was correlated to survival (*P*-values). Figures in bold denote significant values..

**Table 2:** Interrelationships between clinicopathological characteristics of patients and protein expression/activation with bladder cancer using *χ*^2^-analysis

**Variable**	**Numbers**	**Age**	**T-stage**	**Grade**	**Recurrence**	**Metastases at follow-up**
c-Src nuc (negative/positive)	37/21	0.139	0.814	0.706	0.75	0.159
c-Src cyto (negative/positive)	31/27	0.49	0.515	0.689	0.563	0.563
c-Src mem (negative/positive)	30/28	0.956	0.066	0.024*	0.448	0.416
Y^527^ Src nuc (negative/positive)	56/2	0.763	0.097	0.544	0.198	0.113
Y^527^ Src cyto (negative/positive)	37/21	0.197	0.269	0.706	0.822	0.388
Y^527^ Src mem (negative/positive)	26/32	0.483	**0.021** *	0.011*	0.162	0.078
Y^416^ Src nuc (negative/positive)	30/28	0.557	0.693	0.094	0.416	0.773
Y^416^ Src cyto (negative/positive)	29/29	0.79	0.21	0.109	0.192	0.295
Y^416^ Src mem (negative/positive)	33/25	0.304	0.054	0.005*	0.14	0.001*
Y^861^ FAK nuc (negative/positive)	52/6	0.588	0.825	0.211	0.553	0.79
Y^861^ FAK cyto (negative/positive)	38/20	0.602	0.936	0.295	0.569	0.595
Y^861^ FAK mem (negative/positive)	25/33	0.963	0.083	0.125	0.026*	0.045*
Cav nuc (negative/positive)	57/1	0.418	0.245	0.267	0.367	0.267
Cav cyto (negative/positive)	31/27	0.49	0.352	0.689	0.638	0.957
Cav mem (negative/positive)	44/14	0.335	0.09	**0.026**	0.658	0.292
Rho nuc (negative/positive)	29/29	0.425	0.21	0.593	0.601	0.036*
Rho cyto (negative/positive)	30/28	0.956	0.693	0.547	0.814	0.814
Rho mem (negative/positive)	31/27	0.363	0.851	0.689	0.32	0.957

Abbreviations: Cav=caveolin; cyto=cytoplasmic; FAK=focal adhesion kinase; mem=membrane; nuc=nuclear. Figures in bold denote significant values. ^*^Negative correlation.

**Table 3:** Interrelationships between protein markers at the various cellular locations using Pearson's correlation

	***c-Src nuc***	***c-Src cyto***	***c-Src mem***	***Y*^*527*^ *Src nuc***	***Y*^*527*^ *Src cyto***	***Y*^*527*^ *Src mem***	***Y*^*416*^ *Src nuc***	***Y*^*416*^ *Src cyto***	***Y*^*416*^ *Src mem***	***Y*^*861*^ *FAK nuc***	***Y*^*861*^ *FAK cyto***	***Y*^*861*^ *FAK mem***	***Cav nuc***	***Cav cyto***	***Cav mem***	***Rho nuc***	***Rho cyto***	***Rho mem***
*c-Src nuc*																		
PC	—	NS	NS	NS	NS	NS	NS	NS	NS	NS	NS	NS	NS	NS	NS	NS	NS	NS
Sig	—	NS	NS	NS	NS	NS	NS	NS	NS	NS	NS	NS	NS	NS	NS	NS	NS	NS
																		
*c-Src cyto*																		
PC	—	NS	0.5792	NS	NS	NS	NS	NS	NS	NS	NS	NS	NS	NS	NS	NS	NS	0.4922
Sig	—	—	***P*<0.0001**	NS	NS	NS	NS	NS	NS	NS	NS	NS	NS	NS	NS	NS	NS	**0.0001**
																		
*c-Src mem*																		
PC	—	—	—	NS	NS	0.5731	NS	NS	NS	NS	NS	0.5421	NS	NS	NS	NS	NS	0.4939
Sig	—	—	—	NS	NS	***P*<0.0001**	NS	NS	NS	NS	NS	***P*<0.0001**	NS	NS	NS	NS	NS	**0.0001**
																		
*Y*^*527*^ *Src nuc*																		
PC	—	—	—	—	NS	NS	NS	NS	NS	NS	NS	NS	NS	NS	NS	NS	NS	NS
Sig	—	—	—	—	NS	NS	NS	NS	NS	NS	NS	NS	NS	NS	NS	NS	NS	NS
																		
*Y*^*527*^ *Src cyto*																		
PC	—	—	—	—	—	NS	NS	NS	NS	NS	0.4783	NS	NS	NS	NS	NS	NS	NS
Sig	—	—	—	—	—	NS	NS	NS	NS	NS	**0.0001**	NS	NS	NS	NS	NS	NS	NS
																		
*Y*^*527*^ *Src mem*																		
PC	—	—	—	—	—	—	NS	NS	NS	NS	NS	0.6268	NS	NS	NS	NS	NS	NS
Sig	—	—	—	—	—	—	NS	NS	NS	NS	NS	***P*<0.0001**	NS	NS	NS	NS	NS	NS
																		
*Y*^*416*^ *Src nuc*																		
PC	—	—	—	—	—	—	—	NS	NS	NS	NS	NS	NS	NS	NS	NS	NS	NS
Sig	—	—	—	—	—	—	—	NS	NS	NS	NS	NS	NS	NS	NS	NS	NS	NS
																		
*Y*^*416*^ *Src cyto*																		
PC	—	—	—	—	—	—	—	—	NS	NS	NS	NS	NS	NS	NS	NS	NS	NS
Sig	—	—	—	—	—	—	—	—	NS	NS	NS	NS	NS	NS	NS	NS	NS	NS
																		
*Y*^*416*^ *Src mem*																		
PC	—	—	—	—	—	—	—	—	—	NS	NS	NS	NS	NS	NS	NS	NS	NS
Sig	—	—	—	—	—	—	—	—	—	NS	NS	NS	NS	NS	NS	NS	NS	NS
																		
*Y*^*861*^ *FAK nuc*																		
PC	—	—	—	—	—	—	—	—	—	—	NS	NS	NS	NS	NS	NS	NS	NS
Sig	—	—	—	—	—	—	—	—	—	—	NS	NS	NS	NS	NS	NS	NS	NS
																		
*Y*^*861*^ *FAK cyto*																		
PC	—	—	—	—	—	—	—	—	—	—	—	NS	NS	NS	NS	NS	NS	NS
Sig	—	—	—	—	—	—	—	—	—	—	—	NS	NS	NS	NS	NS	NS	NS
																		
*Y*^*861*^ *FAK mem*																		
PC	—	—	—	—	—	—	—	—	—	—	—	—	NS	NS	NS	NS	NS	NS
Sig	—	—	—	—	—	—	—	—	—	—	—	—	NS	NS	NS	NS	NS	NS
																		
*Cav nuc*																		
PC	—	—	—	—	—	—	—	—	—	—	—	—	—	NS	NS	NS	NS	NS
Sig	—	—	—	—	—	—	—	—	—	—	—	—	—	NS	NS	NS	NS	NS
																		
*Cav cyto*																		
PC	—	—	—	—	—	—	—	—	—	—	—	—	—	—	NS	NS	NS	NS
Sig	—	—	—	—	—	—	—	—	—	—	—	—	—	—	NS	NS	NS	NS
																		
*Cav mem*																		
PC	—	—	—	—	—	—	—	—	—	—	—	—	—	—	—	NS	NS	NS
Sig	—	—	—	—	—	—	—	—	—	—	—	—	—	—	—	NS	NS	NS
																		
*Rho nuc*																		
PC	—	—	—	—	—	—	—	—	—	—	—	—	—	—	—	—	0.6326	NS
Sig	—	—	—	—	—	—	—	—	—	—	—	—	—	—	—	—	***P*<0.0001**	NS
																		
*Rho cyto*																		
PC	—	—	—	—	—	—	—	—	—	—	—	—	—	—	—	—	—	0.5600
Sig	—	—	—	—	—	—	—	—	—	—	—	—	—	—	—	—	—	***P*<0.0001**
																		
*Rho mem*																		
PC	—	—	—	—	—	—	—	—	—	—	—	—	—	—	—	—	—	—
Sig	—	—	—	—	—	—	—	—	—	—	—	—	—	—	—	—	—	—

Abbreviations: Cav=caveolin; cyto=cytoplasmic; mem=membrane; nuc=nuclear; NS=non-significant *P*-values; PC=Pearson's correlations; Sig=significance (2-tailed). Figures in bold denote significant values.
